# The Heritability of Amyotrophic Lateral Sclerosis in a Clinically Ascertained United States Research Registry

**DOI:** 10.1371/journal.pone.0027985

**Published:** 2011-11-22

**Authors:** Thomas S. Wingo, David J. Cutler, Nicole Yarab, Crystal M. Kelly, Jonathan D. Glass

**Affiliations:** 1 Atlanta Veterans Administration Medical Center, Atlanta, Georgia, United States of America; 2 Department of Neurology, Emory University School of Medicine, Atlanta, Georgia, United States of America; 3 Department of Human Genetics, Emory University School of Medicine, Atlanta, Georgia, United States of America; National Institutes of Health, United States of America

## Abstract

**Background:**

The genetic basis of amyotrophic lateral sclerosis (ALS) is not entirely clear. While there are families with rare highly penetrant mutations in Cu/Zn superoxide dismutase 1 and several other genes that cause apparent Mendelian inheritance of the disease, most ALS occurs in families without another affected individual. However, twin studies suggest that all ALS has a substantial genetic basis. Herein, we estimate the genetic contribution to ALS in a clinically ascertained case series from the United States.

**Methodology/Principal Findings:**

We used the database of the Emory ALS Center to ascertain individuals with ALS along with their family histories to determine the concordance among parents and offspring for the disease. We found that concordance for all parent–offspring pairs was low (<2%). With this concordance we found that ALS heritability, or the proportion of the disease explained by genetic factors, is between 40 and 45% for all likely estimates of ALS lifetime prevalence.

**Conclusions/Significance:**

We found the lifetime risk of ALS is 1.1% in first-degree relatives of those with ALS. Environmental and genetic factors appear nearly equally important for the development of ALS.

## Introduction

Amyotrophic lateral sclerosis (ALS, also known as motor neuron disease or Lou Gehrig's disease) is a progressive and fatal degenerative illness of motor neurons. The genetic basis of ALS is not known; however, familial clustering of ALS has suggested a genetic basis for the disease. In population-based studies, between 1.6% and 5.6% of individuals with ALS have a relative with ALS [1], [2]. Families with ALS frequently show autosomal dominant transmission, although autosomal recessive pedigrees are also described. Nearly 20% of individuals with familial ALS carry mutations in the gene Cu/Zn superoxide dismutase 1 (SOD1) [3]. Mutations in other genes have also been described [4], [5]. Thus it is clear that some ALS cases have a strong genetic basis, but these families account for fewer than 10% of all ALS cases. However, the exact degree inherited genetic factors play in development of ALS overall remains an open question.

Two studies have addressed this question using concordance for ALS among twins, but the conclusions may be limited by the small numbers of patients included and overlapping samples [6], [7]. The British motor neuron disease (MND) twin study found ALS heritability to be between 38 and 85%, using 76 twin pairs [7]. A more recent study included 171 twin pairs by combining the British MND data with data from twins identified via an ALS registry in the United Kingdom and from the National Swedish Twin Registry [6]. This study found the heritability of ALS to be 76% (95% confidence interval, 60–86%) when individuals with familial forms (i.e. known ALS in other non-twin family members) of the disease were included. However, when familial cases were excluded, the heritability of ALS dropped to 61% (95% confidence interval, 38–78%).

Here we approached the heritability of ALS by examining the concordance among ALS patients (probands) and their parents, using a clinically ascertained population from a single ALS clinical center. This approach is not traditional since we are ascertaining offspring and examining the risk among their parents; however, it is mathematically equivalent to the traditional method of parent-offspring heritability studies where the parent is the proband and allows larger numbers of probands to be examined, enhancing the power of the analysis. Additionally, unlike concordance among twins, concordance among parents and offspring is unlikely to change with time since the majority of parents are deceased by the time their offspring develop ALS, given the mean age of ALS onset is approximately 60 years old [8], [9], [10]. Because the worldwide annual incidence is fairly uniform at approximately 1–2.5 cases per 100,000, (with the notable exception of a historical higher incidence in Guam thought to be due to unique environmental influences in that region [11]), we were able to estimate ALS heritability for all plausible estimates of ALS prevalence. These estimates also take into consideration the slight male predominance of ALS [9], [10], [12].

## Methods

### Objective

Our objective was to estimate the heritability of ALS using the prevalence among relatives ascertained from the Emory ALS Clinic in the United States.

### Participants

Probands were ascertained from a case series database collected by the Emory ALS Center from December 1997 to March 2011. All individuals completed a standardized history that included detailed questions on family history of ALS, a thorough neurologic and neuromuscular examination, and/or electrophysiologic investigation. Family history was obtained both by a questionnaire and verified at the initial interview by a clinician. Inclusion criteria were the diagnosis of ALS and non-missing parental family history data. Only the first identified family member was included when multiple affected individuals in a family were available.

### Concordance for ALS among Relatives

Participants indicated whether their parents had ALS. When possible the affected parents were examined at the Emory ALS Center. Alternatively, the medical records, including physician notes and autopsy records, were obtained to establish the diagnosis in the parents. We defined a concordant parent-offspring pair as a proband with clinical ALS and a parent with ALS as determined using the best available evidence.

### Estimating ALS Heritability

We determined sex–specific concordance rates among all combinations of parent–offspring pairs (i.e., father–daughter, father–son, mother–daughter, and mother–son). To estimate ALS heritability, we followed the methods of Falconer [13], and assumed the existence of a normally distributed latent liability to ALS in the general population. Liability in this model refers to all possible environmental and genetic influences on the development of ALS; individuals beyond a certain liability threshold develop ALS, while those below the threshold do not. The threshold was set using the lifetime prevalence of ALS for the general population. For our model this is the lifetime ALS prevalence among the parents' generation. Since we do not know the lifetime prevalence of ALS in the general population, i.e. among the parents' generation, we first estimated heritability for all likely values of ALS lifetime prevalence taken from the literature, and then came up with point estimates from widely cited prevalence estimates of ALS.

### Statistical Analysis

Heritability was calculated for each combination of parent and offspring genders independently. First, we used our estimate of the lifetime prevalence of ALS for each gender to set the liability threshold for that disease (x) and mean deviate of affected individuals (a). The threshold and mean deviate were calculated for each sex-specific parent-offspring pair. A parent-offspring regression coefficient (b) (half heritability) was obtained using the following equation [13]:
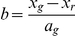
Here the subscripts g and r refer to the sex-specific prevalence from the general population (i.e., proband's sex) or relative (i.e., parent's sex), respectively. The sampling variance of the regression coefficient [13] is given by:
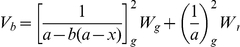
W refers to the prevalence, and the subscripts g and r refer to the sex-specific prevalence from the general population or relative, respectively. Heritability (h^2^) for parent-offspring pairs is [13]:
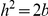
The standard error of heritability is [13]:
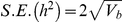
Mean heritability and standard error were calculated weighted by the reciprocal of the sampling variance for each parent-offspring pair.

### Ethics

The Emory University Institutional Review Board approved of the study, and all participants gave informed consent to be in the Emory Clinical Research in Neurology Registry.

## Results

From the Emory ALS research database we ascertained 1088 probands with ALS and parental data. Fourteen probands were excluded from analysis because of lack of information about parental medical history. The demographics of the study population are given in table 1. The demographic characteristics of our ALS population was similar to that reported from other case series and population-based studies [8], [9] with male predominance of 1.45∶1, and a mean age of onset of 58 years old.

**Table 1:** Demographics.

	n = 1088
Sex	
Female (%)	452 (41.5)
Male (%)	636 (58.5)
Race^1^	
American Indian (%)	1 (0.2)
Asian (%)	6 (1.2)
Black (%)	80 (15.7)
White (%)	421 (82.9)
Mean Age of Onset (years ± SD)^2^	58±12.7
First Symptoms^3^	
Bulbar (%)	279 (27.5)
Diaphragm or Other (%)	11 (1.1)
Lower Extremity (%)	364 (35.9)
Upper Extremity (%)	361 (35.6)

1 Racial data collected was collected from 2007 and is present for 47.7% of the cohort. Percentages based on 508 individuals with available data.

2 9.4% of probands had insufficient data to establish an age of onset.

3 6.6% of probands had insufficient data to establish where their first symptoms began. Percentages were based on the total of 1015 individuals.

Among the entire cohort, 45 individuals (4.1%) had a family history of ALS. However, only 24 patients (2.2%) had an affected parent. Interestingly, three individuals had affected siblings and no affected parents, one had an affected child and a second-degree relative, and the remaining 17 had second-degree relatives affected. The parent-offspring concordance is shown by gender for all probands in table 2. For all parent-offspring pairs, the concordance was less than 2%. Concordance was least (0.5%, or 1 of 452 parent-offspring pairs) among fathers of affected women.

**Table 2:** Parent-Offspring Concordance for ALS.

	Father		Mother	
	Percent Concordance	Concordant/Total	Percent Concordance	Concordant/Total
Female Proband	0.22%	1 / 452	1.8%	8 / 452
Male Proband^1^	1.1%	7 / 635	1.3%	8 / 636

1 Missing paternal data on one male proband's father; thus the totals for Male Proband–Father and Male Proband–Mother pairs are not equal.

**Figure 1:**
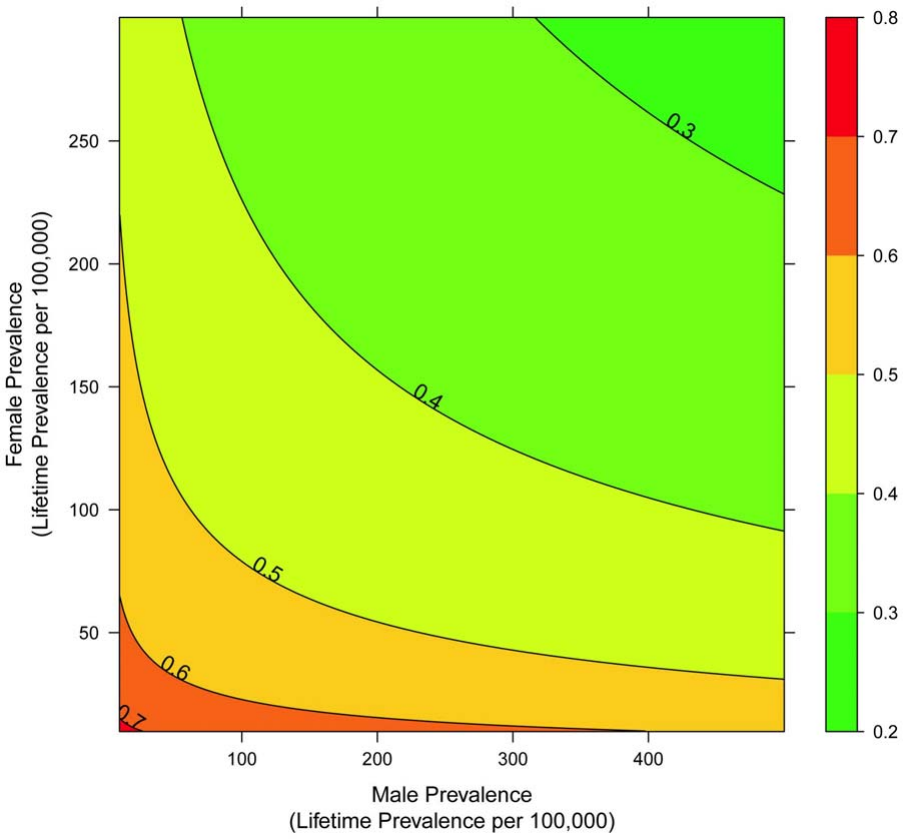
Heritability of Amyotrophic Lateral Sclerosis. A contour plot of the ALS heritability as a function of male and female lifetime ALS prevalence. The bar on the right shows the color code for the corresponding heritability value. The black lines on the graph show the boundaries for heritability values given by the corresponding number above the line.

To estimate the heritability of a trait, we must know the prevalence of the trait among relatives and the general population. Since we do not know the prevalence of ALS in the general population with certainty, we estimated the heritability for ALS using a range of likely male and female ALS prevalence values. We found that for all lifetime prevalence values less than 500 per 100,000 for men and 300 per 100,000 for women, ALS heritability is between 27 and 73%. These results are graphically displayed in figure 1. Next, we estimated ALS heritability for likely values of ALS lifetime prevalence in the United States, assuming an average lifespan of 70 years for men and 75 years for women for our cohort's parents. From the literature we found estimates for ALS lifetime prevalence for men and women that ranged from 150–250 per 100,000 for men aged 70 years and 80–220 per 100,000 for women aged 75 years [14]. Using these values we calculated ALS heritability to be between 36 and 48%. We found a slightly higher ALS heritability of 50.8% (95% confidence interval, 41.4–60.2%) by calculating the lifetime prevalence from the estimated annual incidence rate of ∼2.0 per 100,000 in the United States [9], [15]. These analyses lead us to conclude that genetic factors contribute between 40 and 60% to the risk for developing ALS. To refine these estimates we note that under a polygenic model of disease, heritability should be equal for each pair of parent and offspring; however, we found that the observed value of father-daughter pairs is significantly lower than expected for any heritability above 45% (p = 0.01), implying 45% is an upper bounds for ALS heritability.

To examine the potential confounding role of ethnicity in our cohort, we estimated the heritability among self-identified white individuals alone. This group was chosen because it comprises the majority (82.9%) of the individuals for whom we have ethnicity data. Using the lifetime ALS prevalence of 200 per 100,000, we found ALS heritability is 58.1% (95% CI: 45.0–71.2%), similar to what we found for the entire cohort.

## Discussion

We assessed the risk of ALS among parents of affected individuals in a large case series ascertained from the Emory ALS Center. We found that the risk for ALS among first-degree relatives is 1.1%, which is similar to two other studies that estimated parent-offspring risk by examining children of affected individuals [16], [17] and implies the relative risk among first-degree relatives is 2.2–6.9, depending on the true lifetime prevalence of ALS in the United States. Our data also suggests that inherited factors account for roughly half of the liability for developing ALS, assuming no shared environmental factors. Surprisingly, we found significantly (p = 0.01) fewer concordant female probands and father pairs than expected for heritability values above 45%. There is no simple genetic model that explains this finding (e.g., autosomal dominant, recessive, X-linked, or mitochondrial), and under the polygenic model, this group should have the highest proportion of concordant parent-offspring pairs (instead of the lowest), because ALS is more prevalent in males than females [13]. Recall that in our experiment the probands are the offspring. Thus, father-daughters pairs should exhibit the highest concordance because the risk of ALS is lower in women, causing affected daughters to be shifted further from the mean female liability for ALS than affected sons are from the mean male liability. The consequence of affected daughters having a higher liability is that her parents are more likely to have had ALS, particularly fathers since the risk for ALS is higher in men. While any conclusions from these data must be tempered by the relatively small number of affected parent-offspring pairs, these data do suggest that either the heritability of ALS is less than or equal to 45%, or that ALS is not a significantly polygenic disease. Indeed, there is evidence from the literature that there may be relatively few genetic causes that contribute to ALS considering the recent identification of the hexamer repeat in C9ORF72 and well-described SOD1 mutations that account for a substantial proportion of familial and sporadic ALS cases [4], [18], [19]. However, we find no convincing alternative to the polygenic model of disease based on the observed concordance among our cohort. Other plausible explanations are that people generally know less about the medical health history of their father than their mother, which is suggested by the general trend of lower father-offspring concordance for both male and female probands observed in the data, or that our case series is not reflective of ALS in the general population. However, we note that our population has a similar mean age of onset and prevalence of familial ALS as reported for other population-based ALS studies [1], [8]. In our view, the most likely explanation of the data is simply that the heritability of ALS is between 40 and 45%. This is best seen in figure 1, which shows ALS heritability for all plausible values of ALS lifetime prevalence. The two prior European studies found a higher point estimate of ALS heritability, but our estimate of 40% is contained within their 95% confidence intervals [6], [7]. The different values may simply reflect a true difference between the relative contribution of environmental and genetic factors, or differences in lifetime prevalence of ALS between the study locations. In either case, our findings suggest ALS has roughly equivalent contribution from genetic and environmental factors.

Our study has several strengths. First, we analyzed a large cohort of clinically ascertained cases of ALS. Family histories, including pedigrees, were prospectively ascertained on all individuals. The majority of patients had deceased parents and thus concordance for ALS is unlikely to change with time. One possible concern in our study is ascertainment bias of individuals with a stronger family history, *i.e.* the probability of ascertaining a proband with family history of ALS is higher than those without family history. In this scenario, our results would likely deviate from what we would expect under a polygenic model of disease; however, incomplete ascertainment of families with ALS could also lead to acceptance of a polygenic model when it should be rejected [20], [21]. In our view, referral bias (or incomplete ascertainment of families) seems unlikely, considering the low number of familial ALS cases (4.1%) in our cohort that is similar to what is seen in population-based studies of ALS that found 1.6–5.6% familial ALS [1], [2]. Another potential weakness is that we do not know the exact prevalence of ALS in the general population, which we addressed by using a wide range of likely ALS lifetime prevalence values among men and women.

In summary, we found ALS has a moderate genetic basis, with a likely heritability between 40 and 45%. It is noteworthy that in our cohort nearly half of all concordant parent-offspring pairs are due to known SOD1 mutations, probably because patients with a family history of ALS undergo SOD1 mutation screening. This raises the question of how much of ALS heritability is explained entirely by SOD1 mutations, which should be the focus of future investigations.
